# Angiogenesis-Related Immune Signatures Correlate With Prognosis, Tumor Microenvironment, and Therapeutic Sensitivity in Hepatocellular Carcinoma

**DOI:** 10.3389/fmolb.2021.690206

**Published:** 2021-06-28

**Authors:** Yuan Yang, Guozhi Wu, Qiang Li, Ya Zheng, Min Liu, Lingshan Zhou, Zhaofeng Chen, Yuping Wang, Qinghong Guo, Rui Ji, Yongning Zhou

**Affiliations:** ^1^ The First Clinical Medical College, Lanzhou University, Lanzhou, China; ^2^ Department of Gastroenterology, The First Hospital of Lanzhou University, Lanzhou, China; ^3^ Key Laboratory for Gastrointestinal Diseases of Gansu Province, Lanzhou University, Lanzhou, China

**Keywords:** hepatocellular carcinoma (HCC), tumor immune microenvironment (TIME), immune, prognostic, chemosensitivity

## Abstract

**Background:** Hepatocellular carcinoma (HCC) is one of the highly heterogeneous cancers that lacks an effective risk model for prognosis prediction. Therefore, we searched for angiogenesis-related immune genes that affected the prognosis of HCC to construct a risk model and studied the role of this model in HCC.

**Methods:** In this study, we collected the transcriptome data of HCC from The *Cancer* Genome Atlas (TCGA) and the International *Cancer* Genome Consortium (ICGC) database. Pearson correlation analysis was performed to identify the association between immune genes and angiogenesis-related genes. Consensus clustering was applied to divide patients into clusters A and B. Subsequently, we studied the differentially expressed angiogenesis-related immune genes (DEari-genes) that affected the prognosis of HCC. The most significant features were identified by least absolute shrinkage and selection operator (LASSO) regression, and a risk model was constructed. The reliability of the risk model was evaluated in the TCGA discovery cohort and the ICGC validation cohort. In addition, we compared the novel risk model to the previous models based on ROC analysis. ssGSEA analysis was used for function evaluation, and pRRophetic was utilized to predict the sensitivity of administering chemotherapeutic agents.

**Results:** Cluster A patients had favorable survival rates. A total of 23 DEari-genes were correlated with the prognosis of HCC. A five-gene (including BIRC5, KITLG, PGF, SPP1, and SHC1) signature-based risk model was constructed. After regrouping the HCC patients by the median score, we could effectively discriminate between them based on the adverse survival outcome, the unique tumor immune microenvironment, and low chemosensitivity.

**Conclusion:** The five-gene signature-based risk score established by ari-genes showed a promising clinical prediction value.

## Introduction

As a global health problem throughout the world, hepatocellular carcinoma (HCC) is a highly heterogeneous disease and the third leading cause of tumor-related deaths in cancers (Lafaro et al., 2015). In developing countries, hepatitis B and hepatitis C viruses account for 60 and 33% in the etiology of HCC, respectively, compared with 23 and 20% in developed countries (Parkin, 2006). Besides, alcoholic cirrhosis (Medavaram and Zhang, 2018), non-alcoholic fatty liver disease (NAFLD) (Younossi et al., 2016), and hereditary hemochromatosis (HH) (Cauza et al., 2003) have also been regarded as the risk factors of HCC. These complex factors make the treatment and prognosis of HCC formidable tasks. In addition to surgical procedures, targeted therapy with sorafenib and chemoembolization are now the primary treatments for advanced HCC (Li et al., 2021). With the rapid development of medical technology, the systemic treatment strategy contributed more to improve the prognosis of HCC patients (Anwanwan et al., 2020). However, due to the later detection and high recurrence rate of HCC, nearly 30% or less patients have the opportunity of undergoing a comprehensive treatment leading to worse prognosis (Dufour et al., 2013; Sberna et al., 2018). Some survival prediction models have been constructed with clinical baseline data and tumor biomarkers of HCC with poor accuracy (AlSalloom, 2016). With the progress of genomics technology, the exploration of prognostic gene signatures in HCC has shown broad prospects. Accurate evaluation tools could not only improve the prognosis of HCC patients but also maximize the benefits of chemo- or immunotherapy. Therefore, the exploration of clinical decision–making models is urgently needed.

Angiogenesis has been characterized as an essential process in tumorigenesis because adequate metabolic supply and nutrients are indispensable to promote tumor growth (Folkman, 1972; Hanahan and Folkman, 1996; Morikawa et al., 2002; Berger et al., 2005; Hanahan and Weinberg, 2011). Apart from angiogenesis-inducing agents, numerous genes have also been proven to be modulators of angiogenesis, such as the vascular endothelial growth factor family, hypoxia-inducible factors, and fibroblast growth factors (Ferrara, 2009). The VEGF family has been firstly determined as a set of core molecules in angiogenesis. VEGF-A to -E bound to three tyrosine kinase receptors (VEGFR-1 to -3) and resulted in dimerization and activation of the downstream signaling cascade. Besides, functional polymorphism in VEGF-A has also shown significant correlation with risk of some cancers (Qin et al., 2014). FGF-2, as the activators of angiogenesis, could stimulate new vessels to generate and stabilize (Zhao and Adjei, 2015). These factors contribute to the formation of neo-vasculature in the tumor immune microenvironment (TIME), and the characteristics of the immune contexture significantly influence the outcome of prognosis and therapy (Zhang et al., 2019). However, whether these angiogenesis-related immune signatures could predict the outcome of prognosis and therapy in HCC patients is still unknown.

In our study, we first constructed a multigene risk-score model based on the TCGA cohort and validated it in the ICGC cohort. Subsequently, KEGG enrichment analysis was performed to explore the underlying mechanisms. In addition, tumor immune infiltration was evaluated by single-sample gene set enrichment analysis (ssGSEA). Finally, we further explored the sensitivity of chemotherapeutic agents based on the R package pRRophetic.

## Materials and Methods

### Data Collection From TCGA-LIHC Cohort and ICGC (LIRI-JP) Cohort

**FIGURE 1:**
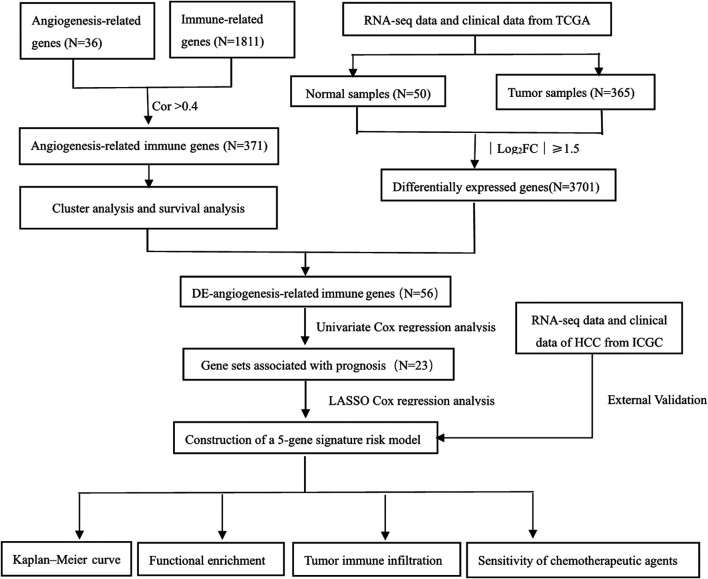
Flowchart of this study.

The transcriptome data and corresponding clinical data of 371 HCC patients were downloaded from TCGA-LIHC as the discovery cohort (https://portal.gdc.cancer.gov). Five samples with the survival time of 0 were excluded. Similarly, the ICGC dataset with another 231 HCC patients (https://dcc.icgc.org/projects/LIRI-JP) was obtained as a validation cohort. A list of recognized angiogenesis-related genes and immune-related genes was downloaded from the MSigDB (http://software.broadinstitute.org/gsea/msigdb) and ImmPort database (http://www.immport.org), respectively. The flowchart is shown in Figure 1.

### Cluster Analysis Based on Angiogenesis-Related Immune Genes

The Pearson correlation coefficient was utilized to identify the correlation between angiogenesis-related genes and immune-related genes. In this analysis, the parameter r fluctuating from 0.4 to 0.6 had moderate correlation. *p* < 0.001 was statistically significant. Therefore, the immune genes with correlation coefficients more than 0.4 and *p*-value less than 0.001 were considered angiogenesis-related immune genes (ari-genes). Cluster analysis algorithms were utilized as a tool with the goal of exploring hidden groupings in a large dataset and frequently used in exploratory public data analysis in recent years. The principle of these algorithms was to form several groupings in such a way that data within a cluster have a higher measure of similarity. Therefore, a consensus clustering analysis was further performed based on the R package ConsensusClusterPlus. To evaluate the prognostic implication of ari-genes in the TCGA cohort, the Kaplan–Meier survival curve was subsequently plotted to compare the OS of the different subgroups.

### Construction and Validation of Risk Model Based on DEari-Genes Affecting Prognosis

In order to develop more powerful risk models, the R package limma was utilized to identify the differentially expressed angiogenesis-related immune genes (DEari-genes) with the threshold of a false discovery rate (FDR) value < 0.05 in the discovery cohort. Univariate Cox regression was performed to screen OS-related DEari-genes. An interaction network for the OS-related DEari-genes was generated by the STRING database (https://string-db.org/). LASSO-penalized Cox regression could improve the accuracy and efficacy of prediction on risk and be widely used in data mining recently (Tibshirani, 1997; Simon et al., 2011). Those genes found to be statistically significant in the univariate Cox regression were then used in the least absolute shrinkage and selection operator (LASSO) algorithm for variable selection and subsequently shrinkage with the R package glmnet. To minimize the risk of overfitting, LASSO regression was performed with tenfold cross validation and run for 1,000 cycles with a random stimulation of 1,000 times to prevent overfitting effects of the model. Next, the ari-genes with the frequency more than 100 times were selected for Cox analysis to construct the benefit model. The risk score of angiogenesis-related immune signatures for each patient was calculated as follows:

$$f\left(x\right)=\sum_{n=1}^{n}\left(\text{expression}\;\text{level}\;\text{of}\;\text{genes}*\text{regression}\;\text{coefficient}\right).$$

All patients were stratified into high-risk and low-risk groups by the median risk score. Besides, PCA and t-SNE were performed to explore the distribution of different groups using R packages stats and Rtsne, respectively. Finally, the Kaplan–Meier survival curve was plotted to compare the OS of the two groups, and the one-, two-, and three-year ROC curves of the risk model were drawn to evaluate the prognostic performance of the gene signature.

### Functional Enrichment Analysis

To elucidate the potential biological roles that were associated with the established risk score, the DEGs between the high-risk and low-risk groups were utilized to perform enrichment analyses. We first identified the expression of differentially expressed gene (DEG) sets between high–risk score and low–risk score groups. The thresholds were set as |log2FC| >1.5 along with FDR <0.05. Kyoto Encyclopedia of Genes and Genomes (KEGG) analysis was conducted by R software. The R package clusterProfiler was utilized to explore the biological attributes of these DEGs.

### Evaluation of Tumor-Infiltrating Immune Cells

To analyze the immune-cell characteristics between the different risk groups, we used single-sample gene set enrichment analysis (ssGSEA) based on the R package gsva. The immune infiltration statuses and relevant immune-related pathways were calculated among the samples from the TCGA-LIHC and LIRI-JP datasets.

### Exploration of the Sensitivity of Chemotherapeutic Agents

To predict the sensitivity of chemotherapeutic agents, the R package pRRophetic was utilized to measure the half-maximal inhibitory concentration (IC50) of samples in different groups by ridge regression. According to AJCC guidelines, antitumor drugs such as cisplatin, doxorubicin, mitomycin, and sorafenib were selected as candidate agents. The IC50 in different groups was compared by the Wilcoxon signed-rank test subsequently.

### Statistical Analysis

All statistical analysis was conducted in R software 3.6.3. The *p*-value < 0.05 and *p*-value < 0.001 were considered statistically significant and highly significant. FDR <0.05 was considered statistically significant.

## Results

### Cluster Analysis Based on Angiogenesis-Related Immune Genes

**FIGURE 2:**
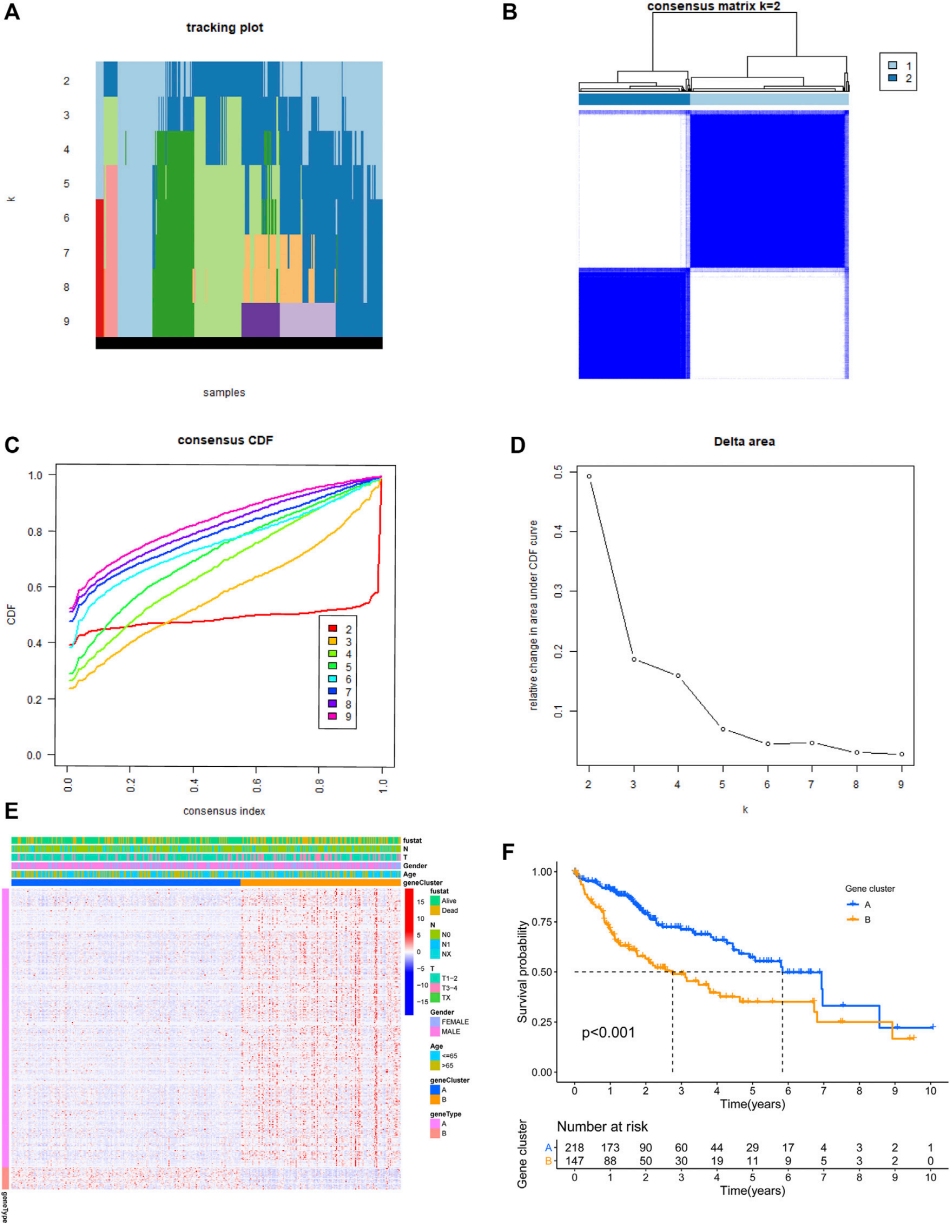
Two clusters based on the expression level of angiogenesis-related immune genes (ari-genes) in the TCGA cohort. **(A)** The sample distribution changed with k valued 2 to 9. **(B)** Relative change in area under the CDF curve with k = 2. **(C)** Consensus clustering cumulative distribution function (CDF) with k valued 2 to 9. **(D)** Consensus clustering matrix for k = 2. **(E)** Heatmap of ari-genes between two clusters in the TCGA cohort. **(F)** Kaplan–Meier survival curves for clusters A and B of the TCGA dataset (*p* < 0.001). A and B represent different immune statuses.

In order to identify ari-genes, Pearson correlation coefficient analysis was conducted. This analysis screened out 371 ari-genes in the TCGA-LIHC cohort (cor >0.4; Supplementary Table S1). These ari-genes were further utilized for cluster analysis. Most of the samples in this study are concentrated on three different positions (far left, middle, and far right). The density is too high to present every single HCC sample. Therefore, these patients from the discovery group were clustered into two subgroups. As shown in Figures 2A–D, k = 2 was considered the excellent cluster number due to its optimal clustering stability in the TCGA cohort. Subsequently, the heatmap of individual clusters was drawn to show the trend of candidate gene expression (Figure 2E). Finally, the survival analysis was performed and showed the better result. Our result showed that cluster A patients had more favorable overall survival (OS) rates than patients of cluster B (*p* < 0.001; Figure 2F).

### Identification of Ari-Genes With Prognostic Value and Establishment of Prognostic Models

**FIGURE 3:**
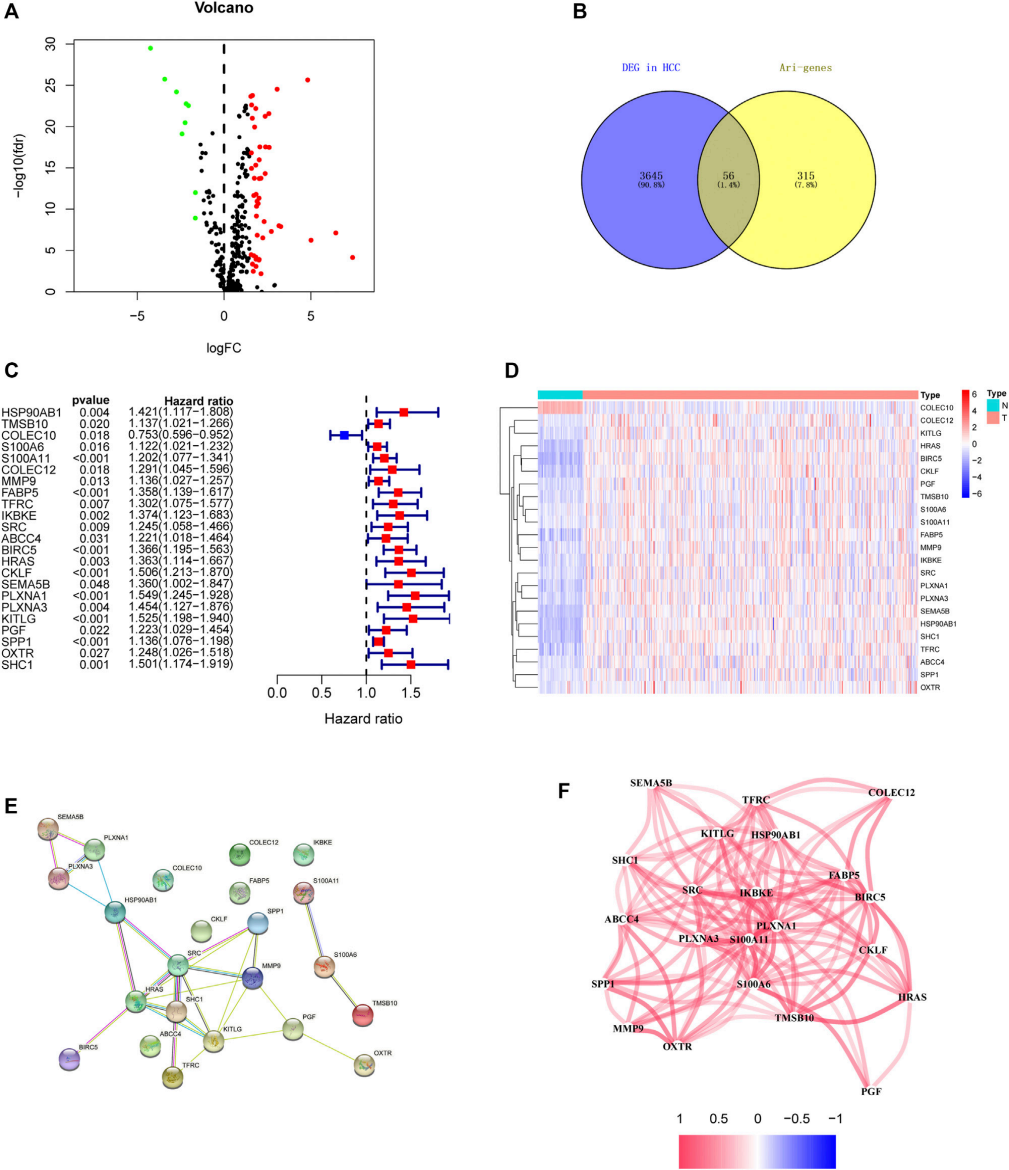
Identification of the candidate angiogenesis-related immune genes in the TCGA discovery cohort. **(A)** Heatmap of DEari-genes between tumor and normal tissues. Red color represents up-regulation of genes, and green color represents down-regulation of genes. **(B)** Volcano plot of DEari-genes between tumor and normal tissues. **(C)** Forest plots showing OS-related ari-genes via univariate Cox regression. **(D)** Heatmap of OS-related ari-genes. **(E)** PPI network indicating the interactions among these candidate genes. **(F)** Network showing the correlation of candidate genes.

**FIGURE 4:**
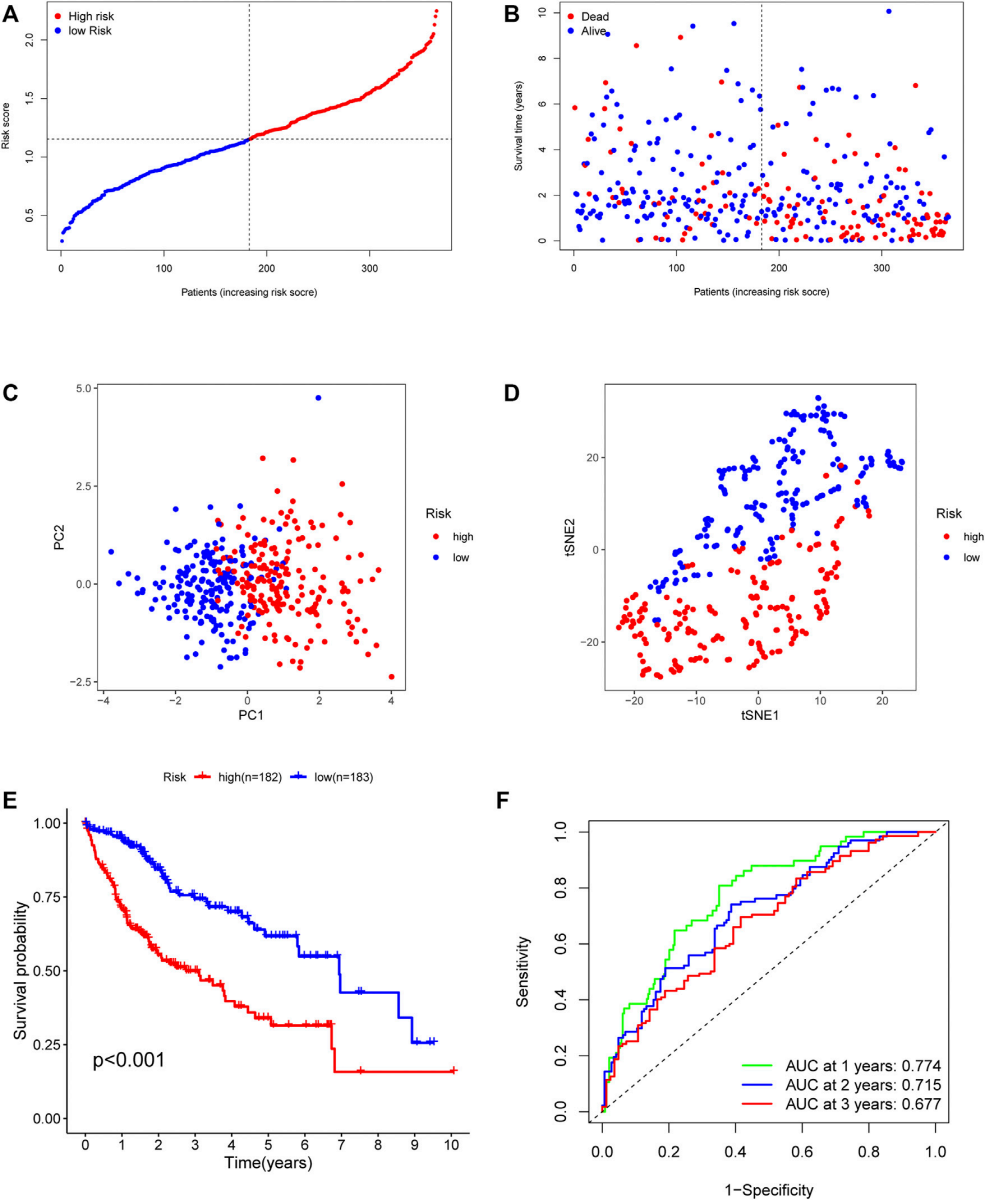
Prognostic value of the five-gene risk model in the TCGA cohort. **(A)** The median value of risk scores with survival and statuses of HCC patients depends on the five-gene risk model in the TCGA cohort. **(B)** The distribution of risk scores with survival and statuses of HCC patients depends on the five-gene risk model in the TCGA cohort. **(C)** Principal component analysis of HCC patients in the TCGA cohort. **(D)** t-SNE analysis of HCC patients in the TCGA cohort. **(E)** Survival analysis of patients in the high-risk group and low-risk group based on the prediction risk score formula. **(F)** One-, two-, and three-year ROC curves of the benefit model for assessing the prognostic performance of the gene signature in the TCGA cohort.

In order to establish powerful predictive models, 56 genes with significantly differential expression were identified as DEari-genes, and univariate Cox regression analysis was conducted to identify OS-related gene sets (Figures 3A,B, Supplementary Table S2). 23 genes were found to have correlation with OS and evaluated between tumor and normal tissues by heatmap (Figures 3C,D). An interaction network for these genes was generated by the STRING database and showed regulation positively with each other (Figures 3E,F). LASSO-penalized Cox regression was performed to further analyze these 23 genes. Five candidate genes were determined and shown in different clusters (Supplementary Figure S1). The risk score formula reads as follows: risk score = 0.165047964281723* mRNA expression level of BIRC5 + 0.135792073795595* mRNA expression level of KITLG + 0.0483865964062503* mRNA expression level of PGF + 0.067693493533674* mRNA expression level of SPP1 + 0.0407522078712915 * mRNA expression level of SHC1. Based on their risk scores, HCC patients in the training set were divided into high- and low-risk groups (Figures 4A,B). Kaplan–Meier survival analysis was performed, and patients in the high-risk group showed significantly shorter OS than those in the low-risk group (*p* < 0.001) (Figure 4E). Then, the ROC curves were plotted, and the AUC values calculated from TCGA for 1, 2, and 3 years were 0.774, 0.715, and 0.677, respectively (Figure 4F). PCA and t-SNE were further applied to demonstrate the distribution in discrete directions (Figures 4C,D).

### Validation of Prognostic Angiogenesis-Related Immune Signatures With External Dataset

**FIGURE 5:**
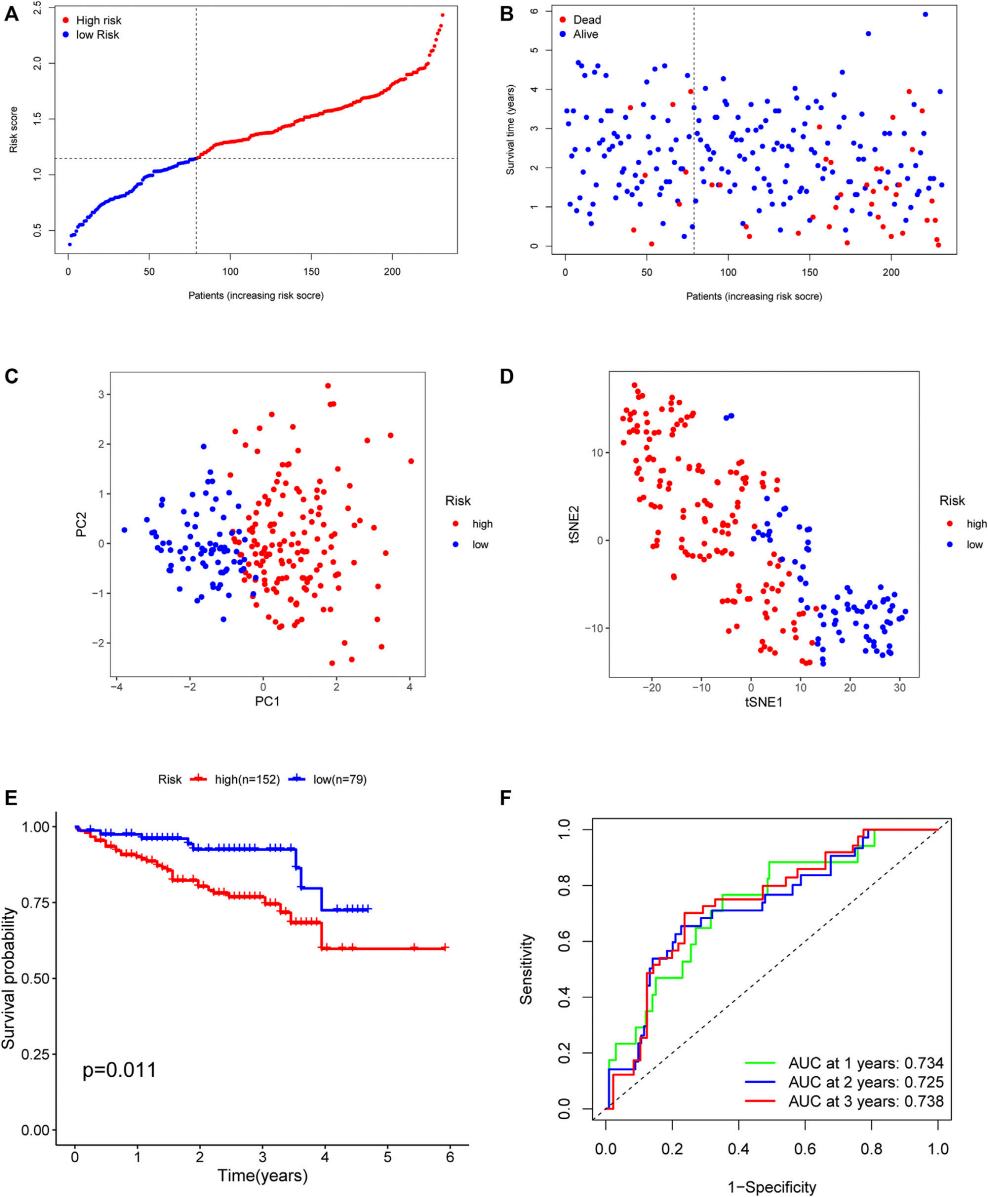
Validation of the risk model in the ICGC cohort. **(A)** The median value of risk scores with survival and statuses of HCC patients depends on the five-gene risk model in the ICGC cohort. **(B)** The distribution of risk scores with survival and statuses of HCC patients depends on the five-gene risk model in the ICGC cohort. **(C)** Principal component analysis of HCC patients in the ICGC cohort. **(D)** t-SNE analysis of HCC patients in the ICGC cohort. **(E)** Survival analysis of patients in the high-risk group and low-risk group based on the prediction risk score formula. **(F)** One-, two-, and three-year ROC curves of the benefit model for assessing the prognostic performance of the gene signature in the ICGC cohort.

To evaluate the predictive value of the identified angiogenesis-related immune signatures from the discovery set, the ICGC dataset was introduced as the validation group. The same formula as that from the TCGA cohort was used to calculate the risk score of each patient in the validation group (Figures 5A,B). As shown in Figure 5E, the patients in the high–risk score group had a reduced survival time compared to those in the low–risk score group. Besides, the validation results showed that the AUC of the angiogenesis-related immune signatures was 0.734 in 1 year, 0.725 in 2 years, and 0.738 in 3 years (Figure 5F). Similarly, PCA and t-SNE analysis showed the same results as those in the TCGA cohort (Figures 5C,D).

### Comparison of the Five-Gene Risk Model and Other Models

Next, we compared the performance of our established risk model with those of four other prognostic models: the seven immune-related–gene signature (Liu et al., 2020), the twelve-gene signature (Ouyang et al., 2020), the HCC prognostic evaluation model (Zhang et al., 2020), and another HCC immune signature (Pan et al., 2020) published in recent years (Table 1).

**TABLE 1:** Comparison of the risk model and other models.

Study	Signature	AUCs in the training set	AUCs in the validation set
Our study	5-Gene	0.774, 0.715, and 0.677 (TCGA N = 365)	0.734, 0.725, and 0.738 (ICGC N = 231)
Liu et al. (2020)	7-Gene	0.778, 0.754, and 0.742 (TCGA N = 365)	0.717, 0.636, and 0.616 (ICGC N = 231)
Ouyang et al. (2020)	12-Gene	0.77, 0.73, and 0.72 (TCGA N = 365)	0.630, 0.680, and 0.660 (GEO N = 233)
Zhang et al. (2020)	9-Gene	0.805 (TCGA N = 365)	0.582 (ICGC N = 231)
Pan et al. (2020)	4-Gene	0.700, 0.652, and 0.630 (TCGA N = 365)	No external validation

### Independent Prognostic Value of the Five-Gene Signature

**FIGURE 6:**
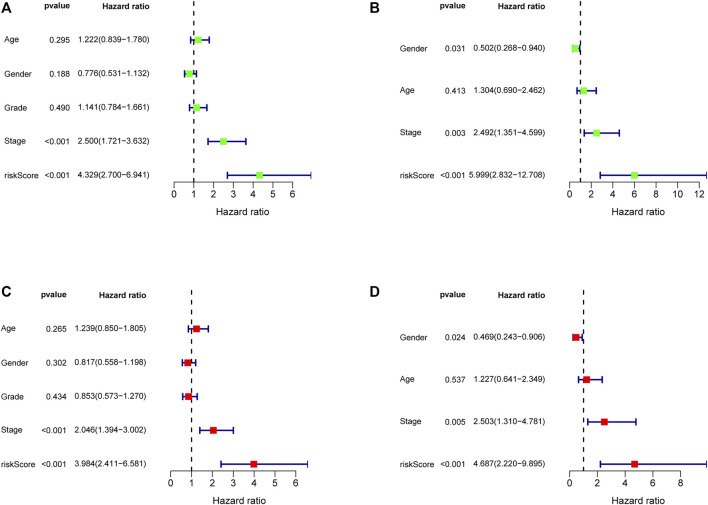
Forest map of univariate and multivariate regression analyses in the TCGA discovery cohort **(A, C)** and the ICGC validation cohort **(B, D)**.

In order to evaluate the independent prognostic predictor for OS, univariate and multivariate Cox regression analyses were carried out successively. We demonstrated that the stage (*p* < 0.001, HR = 2.500, 95% CI [1.721–3.632]) and risk score (*p* < 0.001, HR = 4.329, 95% CI [2.700–6.941]) were significantly associated with OS in the TCGA cohort (Figure 6A). After correction for other confounding factors, the stage (*p* = 0.003, HR = 2.492, 95% CI [1.351–4.599]) and risk score (*p* < 0.001, HR = 5.999, 95% CI [2.832–12.708]) still showed statistical differences by multivariate Cox regression analysis (Figure 6C). Therefore, the stage and risk score are presented as independent prognostic predictors. The results were verified in the ICGC cohort (Figures 6B,D).

### Functional Analysis of the Angiogenesis-Related Immune Signatures

**FIGURE 7:**
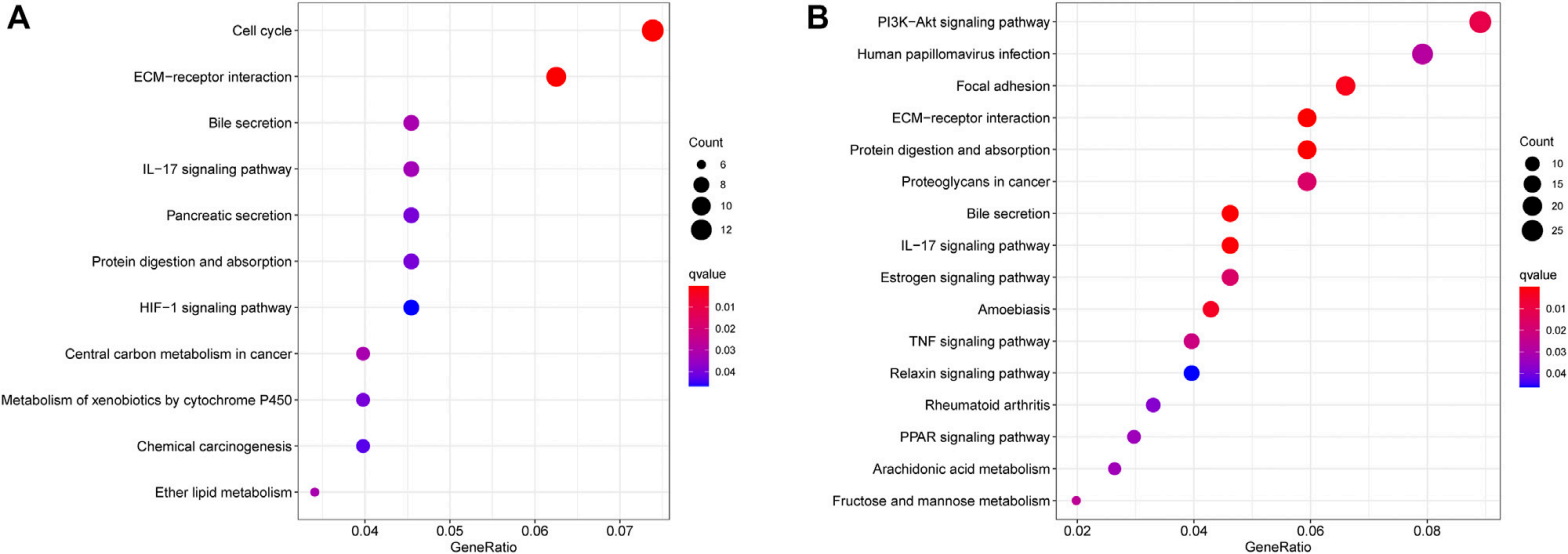
The significant KEGG pathways in the TCGA cohort **(A)** and ICGC cohort **(B)** are displayed.

To elucidate the potential influence of the classifier that was associated with the risk score, we firstly screened DEGs between the high-risk group and the low-risk group. KEGG pathway analyses were further performed to compare the high- and low-risk groups. As expected, KEGG pathway analyses showed that DEGs from TCGA cohorts were mainly involved in several immune-related pathways, such as cell cycle, ECM−receptor interaction, bile secretion, IL−17 signaling pathway, pancreatic secretion, and protein digestion and absorption (Figure 7A). Four pathways were validated by the ICGC cohort, including ECM−receptor interaction, bile secretion, IL−17 signaling pathway, and protein digestion and absorption (Figure 7B). Interestingly, the TIME-associated ECM–receptor interaction was enriched in both cohorts (adjusted *p* < 0.05, Figure 7).

### Evaluation of Tumor Immune Infiltration

**FIGURE 8:**
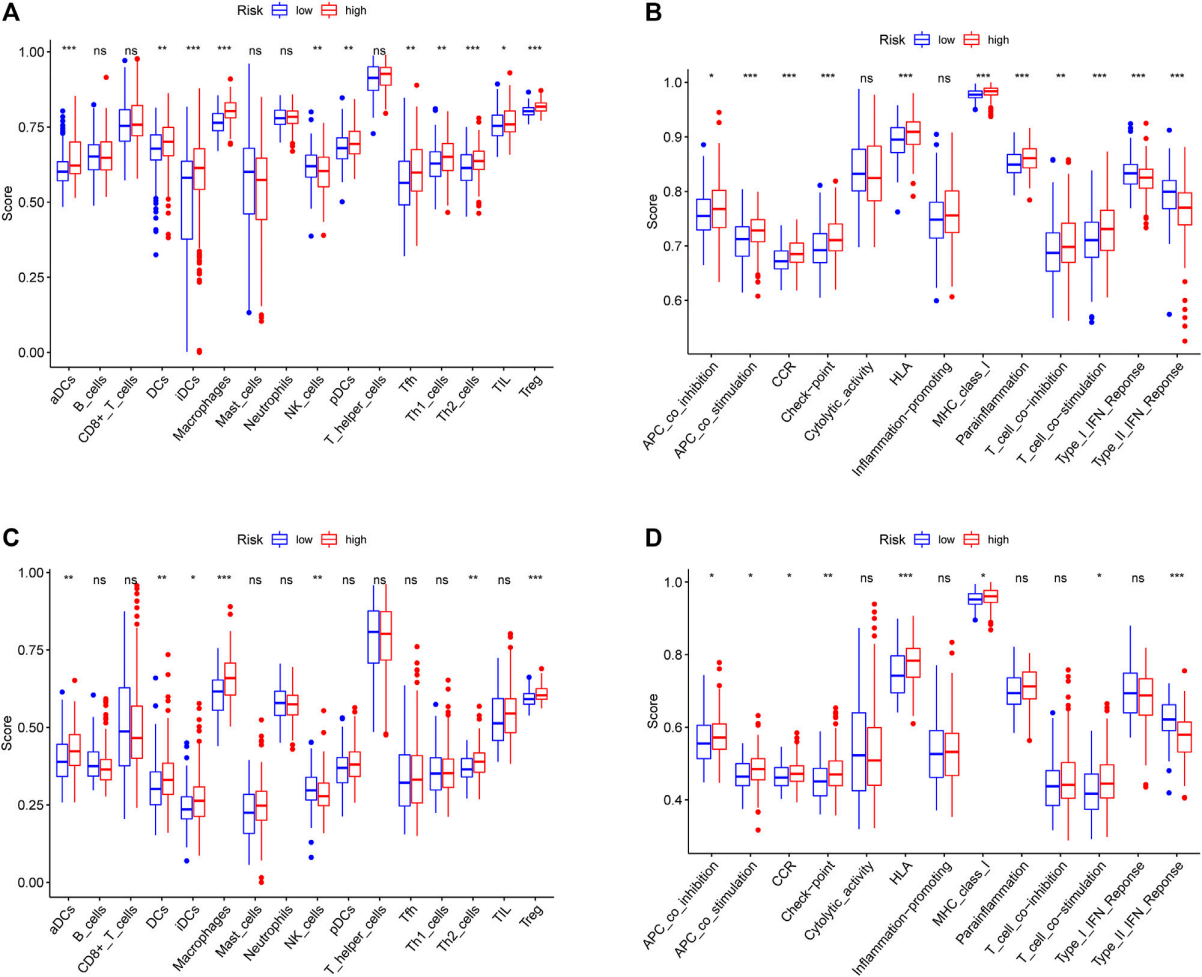
Comparison of the immune status between the high-risk group and the low-risk group in the TCGA cohort **(A, B)** and ICGC cohort **(C, D)**. The difference of 16 immune cells **(A, C)** and 13 immune-related functions **(B, D)** is based on ssGSEA scores. *p < 0.05; **p < 0.01; ***p < 0.001.

To further explore the potential correlation between the risk score and the TIME, we consequently evaluated immune infiltration status among different samples. We revealed that several tumor-infiltrating immune cells were abundant in the high-risk group. In both cohorts, tumor-infiltrating immune cells, including aDCs, DCs, iDCs, Th2 cells, and Treg cells, showed more positive correlation with a higher risk score, implying significant roles of these infiltrating cells in pathogenesis or progression of HCC (all adjusted *p* < 0.05, Figures 8A,C). Interestingly, we could more effectively differentiate between two risk groups in both cohorts based on contents of the antigen presentation process, including aDCs, DCs, iDCs, APC_co_inhibition, APC_co_stimulation, HLA, and MHC_class_I. After reanalysis of the KEGG pathway, we found the ECM−receptor interaction had a relatively higher score in the high-risk group of the TCGA and ICGC cohorts (adjusted *p* < 0.05, Figure 7). Moreover, the scores of APC_co_inhibition, APC_co_stimulation, CCR, Check−point, HLA, MHC_class_I, and T_cell_co_stimulation were higher in the high-risk group, while the activity of type II IFN response was just the opposite (adjusted *p* < 0.05, Figures 8B,D). The result was consistent with the findings of the KEGG analysis.

### Analysis of the Correlation Between the Constructed Risk Model and Common Chemotherapeutics

**FIGURE 9:**
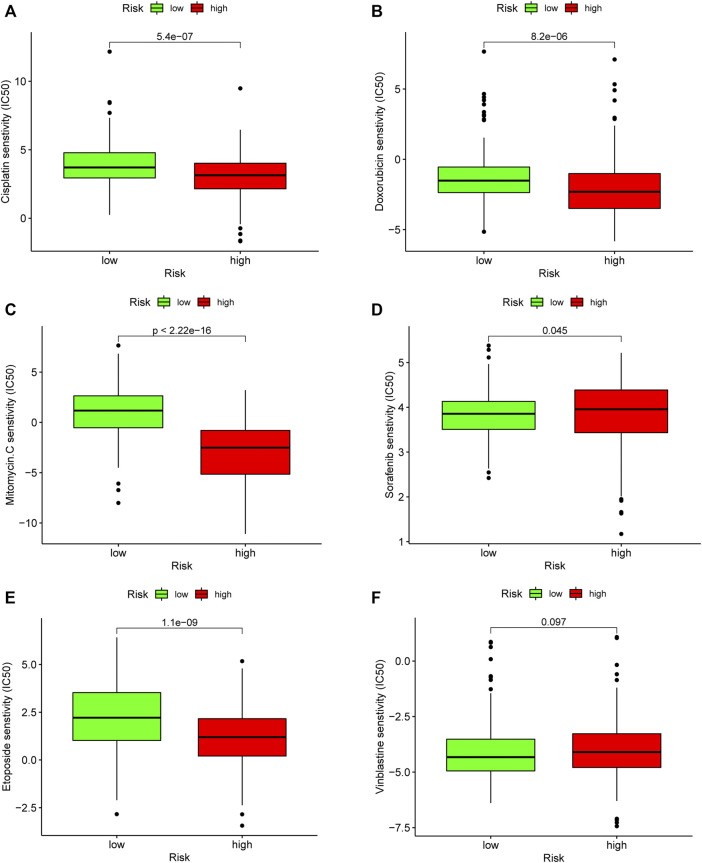
Evaluation of chemosensitivity by the risk model. The model showed high risk scores were associated with a lower IC50 for chemotherapeutics such as **(A)** cisplatin, **(B)** doxorubicin, **(C)** etoposide, and **(E)** mitomycin C, whereas they were related to a higher IC50 for **(D)** sorafenib.

To evaluate the risk model in the clinic for HCC treatment, we attempted to explore associations between risk scores and the efficacy of administering common chemotherapeutics. Our study revealed that a lower risk score was related to higher IC50 among antitumor drugs, such as cisplatin, doxorubicin, etoposide, and mitomycin C, whereas it was associated with a higher chemosensitivity in sorafenib (*p* = 0.045) (Figure 9). Our results indicated that the established model had a potential predictive value for chemosensitivity.

## Discussion

Previous studies have reported expression levels of BIRC5 (Jin et al., 2015), KITLG (Hu et al., 2021), SPP1 (Long et al., 2018), and SHC1 (He et al., 2019) could serve as biomarkers for predicting prognosis in HCC. BIRC5 is essential for cell division and death and promotes the progression of HCC (Wheatley and Altieri, 2019). A previous study has also confirmed that OCT4 could enhance the expression of BIRC5 *via* the inhibition of cell arrest in HCC. This promoted the proliferation of cancer cells and reduced their susceptibility to chemo- and radiotherapy (Su, 2016). KITLG is a ligand of the c-kit tyrosine kinase receptor and found with multiple biological functions in recent years. Aggressive expression of KITLG mediated by the autocrine/paracrine stimulation-loop mechanism has been identified in multiple cancer types such as uveal melanoma (Lefevre et al., 2004), glioma (Sun et al., 2006), breast cancer (Han et al., 2008), and non-small-cell lung cancer (Théou-Anton et al., 2006; Martinho et al., 2008; Levina et al., 2010). However, the roles of KITLG deserve further study in HCC. Secreted phosphoprotein 1 (SPP1) plays a pivotal role in the growth, proliferation, migration, and apoptosis of cancer cells. Interestingly, SPP1 could promote stem-like phenotype in tumorigenesis and further result in chemo-resistance (Liu et al., 2016). Many studies have implicated SHC1 involvement in signaling by epidermal growth factor receptor-2 (HER-2), RAS/MAPK, and PI3K, all of which have a positive effect on tumorigenesis (Das and Vonderhaar, 1996; Fox et al., 2009; Hudson et al., 2014). In recent years, some researchers have proposed that dysregulation of SHC1 might result from extensive epigenetic reprogramming that interferes with normal interactions and solid matrix, mediating metastasis (Terada, 2019). However, the prognosis and roles of PGF have not been reported. These factors were screened out in the univariate Cox regression analysis and found correlated with OS in this study. These results significantly indicated the possibility of constructing a risk model with these ari-genes.

In this study, a risk model based on angiogenesis-related immune signatures was constructed to evaluate the prognosis of HCC patients, immune infiltration status, and drug chemosensitivity to HCC. First, we retrieved raw data of mRNA from the TCGA-LIHC cohort, and the samples with incomplete clinical information were eliminated. Co-expression analysis was performed to classify ari-genes, and the survival curve based on individual clusters showed significant difference. However, the survival curve is crossed, and a great deal of genes limit its clinical application. Therefore, it is necessary to construct an easy-to-use and powerful model. Second, we performed univariate analysis to screen OS-related DEari-genes. These genes were introduced to a modified Lasso penalized regression to determine candidate genes. Third, we calculated each AUC value of ROC at the time of 1, 2, and 3 years to differentiate the high- and low-risk groups among patients with HCC and eventually get the optimal model. The risk model integrating the five-gene signature was further validated in the ICGC cohort. Compared with some previous models, our five-gene risk model showed better performance in the evaluation of prognosis value, with the AUC value of 0.774. Besides, the stage and risk score are presented as independent prognostic predictors. The results were verified in the ICGC cohort. Fourth, we evaluated this novel model under tumor-infiltrating immune cells and chemotherapy. Our model proved to be significant in differentiating between high and low chemosensitivity to HCC. Thus, the present study provides a more precise tool in clinical decision-making.

The tumor immune microenvironment (TIME) has been proven to exert important effects on the treatment response (Teng et al., 2015; Li et al., 2020). Various immune cells might function as a tumor inhibitor or promoter and play a potential role in the regulation of HCC (Lei et al., 2020). Mounting data suggest that angiogenesis is involved in the interactions among tumor cells, various tumor-related stromal cells, and their bioactive products, which revealed that pathological angiogenesis was regulated in a variety of ways (Balkwill et al., 2012; De Palma et al., 2017). Tumor-associated macrophages (TAMs) have been proven to mediate angiogenesis by secreting growth factors and inflammatory factors, thereby activating vascular cell proliferation (De Palma et al., 2017). Regarding the regulatory function of lymphocytes, some evidence showed that T cell subsets (Th1, Treg (Motz and Coukos, 2011) and CD4^+^ Th2 cells (DeNardo et al., 2009)) could also play pro-angiogenesis roles through different mechanisms. Previous studies showed that poor prognosis of cancer patients is greatly correlated with the proportion of M2-like TAMs (Ni et al., 2019; Yan et al., 2020; Wang et al., 2021; Ye et al., 2021). Therefore, whether high risk score is positively correlated with M2-like macrophages needs to be further confirmed. Besides, NK cells (Bruno et al., 2014) and DC cells modulated vascularization directly or indirectly. Interestingly, we could more effectively differentiate between two risk groups in both cohorts based on contents of the antigen presentation process. Some studies indicated that DCs could stimulate some specific T cell responses and further kill a bit more cancer cells *via* the antigen presentation process (Zhou et al., 2019). In this study, we found several tumor-infiltrating immune cells, including aDCs, DCs, iDCs, Th2 cells, and Treg cells, showed more positive correlation with a higher risk score. DCs and T cell subsets were mainly enriched in the high-risk group, which is consistent with that reported in the previous studies. Dendritic cells (DCs) are the main regulators of immune tolerance or response and could enhance the efficacy of immune check-point inhibitors in DC-dependent ways (Martinek et al., 2019). Besides, DCs and macrophages are responsible for capturing antigens on MHC-I for activating CD8^+^ T cells and initiating immune responses, thereby overcoming resistance to immunotherapies (Banchereau and Steinman, 1998; Guerriero, 2019). However, in this study, the increased infiltration of DCs was not associated with higher proportions of CD8^+^ T cells in the high-risk group, implicating a compromised antigen presentation function in the high-risk group. Besides, the previous study indicated that the increased infiltration of Treg cells correlates with CD8^+^ T cell impairment and adverse survival in HCC patients, which is consistent with present results (Fu et al., 2007). Therefore, despite the antigen presentation correlation with increased co-stimulator and MHC class I expression in the high-risk group, our results indicate that tumor-infiltrating immune cells, including aDCs, DCs, iDCs, Th2 cells, and Treg cells, showed more positive correlation with a higher risk score, implying significant roles of these infiltrating cells in pathogenesis or progression of HCC.

Although the underlying mechanisms of tumor immunity have been studied in the past few years, the potential modulation between tumor immunity and angiogenesis remains elusive (McKelvey et al., 2018). KEGG pathway analysis linked ECM–receptor interaction with immune response. The ECM–receptor interaction signal pathway was involved in progression of various cancers (Andersen et al., 2018; Yan et al., 2018; Bao et al., 2019). As is known to all, the extracellular matrix (ECM) not only forms the skeleton of tissue but also promotes malignant phenotypes, such as maintaining proliferation signals, promoting cell survival, migration, differentiation, and angiogenesis, and regulating immune function (Pickup et al., 2014). The transformation of normal cells of epithelial cells into malignant cells could promote metastasis and mediate poor prognosis, which might be the result of the stiffness of ECM (Grasset et al., 2018; Katara et al., 2018). Interestingly, ECM modification, especially stiffness, was also associated with resistance of chemotherapeutic drugs. ECM stiffness serves as a barrier and impedes the effective uptake and delivery of drugs in the local environment of the tumor (Najafi et al., 2019), which further demonstrates the causes of the resistance of chemotherapies. Meanwhile, our risk model revealed that the high risk was associated with high sensitivity of chemotherapy drugs, such as cisplatin, doxorubicin, etoposide, gemcitabine, and mitomycin C, except for sorafenib and vinblastine. These results could guide chemotherapeutic agents’ decision-making in clinical practice.

To the best of our knowledge, this is the first study identifying prognosis-related ari-genes and developing the risk model of prognosis and chemosensitivity in patients with HCC. However, limitations of this study should be mentioned. First, our risk model had a certain predicative value, but it was constructed and validated with retrospective data from TCGA and ICGC public databases. Some prospective studies are needed to verify its clinical utility. Nevertheless, these public databases are well characterized owing to containing the largest sample size up to now. Second, the relationship between the risk score and immune activity should be experimentally addressed in the future. Furthermore, subsequent studies should focus on the mechanism of drug response and acquired resistance to chemotherapy.

## Conclusion

In summary, we constructed a risk model based on ari-genes to assess prognosis, immune infiltration status, and chemotherapy sensitivity in HCC. This model would support clinical decision-making in evaluation of prognosis and drug treatment.

## Data Availability

The datasets presented in this study can be found in online repositories (The Cancer Genome Atlas (TCGA) and The International Cancer Genome Consortium (ICGC)). The names of the repository/repositories and accession number(s) can be found in the article/Supplementary Material.
