# Supplementary material for: Angiogenesis-Related Immune Signatures Correlate With Prognosis, Tumor Microenvironment, and Therapeutic Sensitivity in Hepatocellular Carcinoma
Source: Front Mol Biosci. 2021 Jun 28;8:690206. doi: 10.3389/fmolb.2021.690206 (PMC8273615; doi:10.3389/fmolb.2021.690206)
Supplement: Supplementary file 1 [file DataSheet4.DOCX]

Supplementary Material

# Supplementary Data

# Supplementary Figures and Tables

## Supplementary Figures


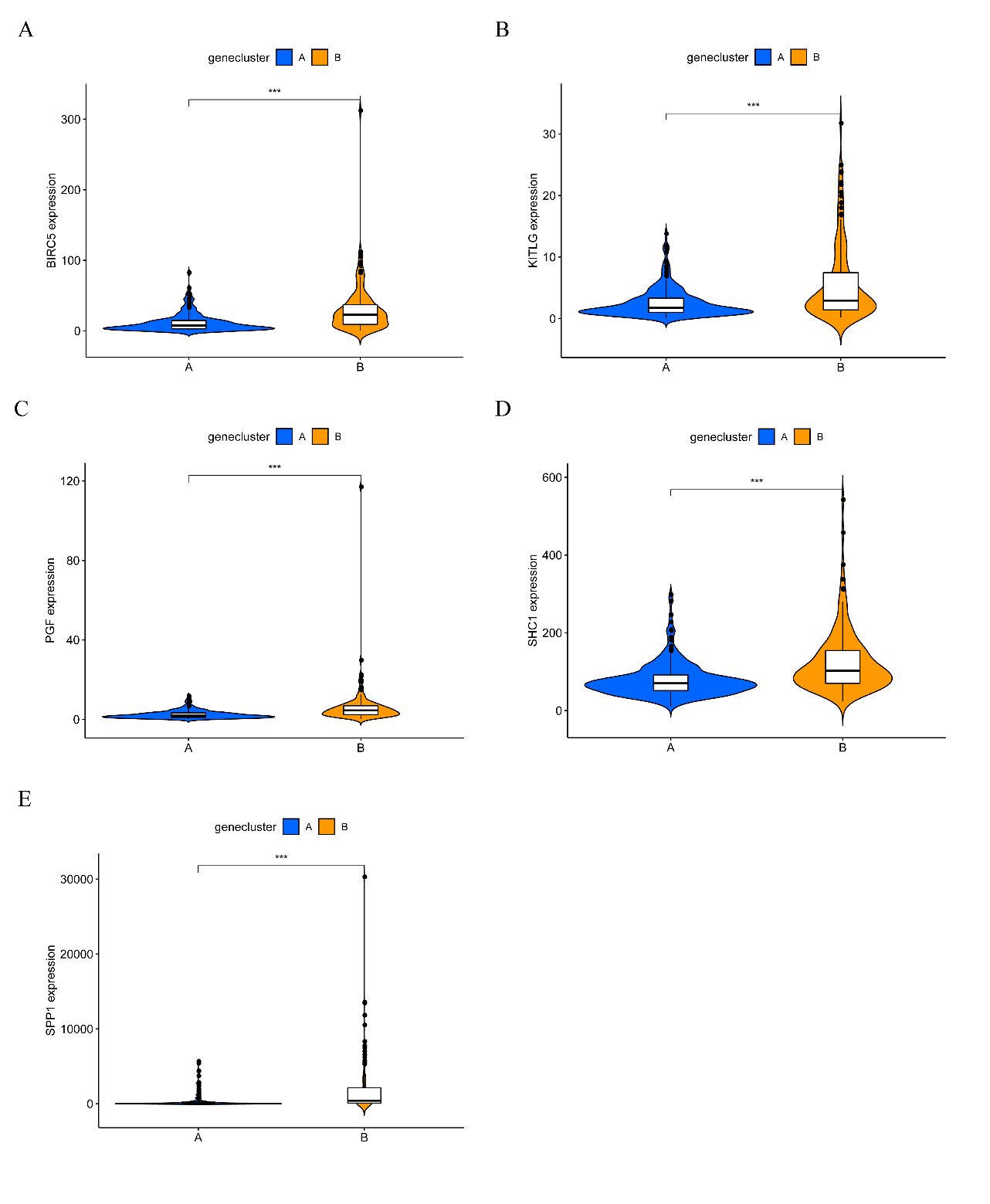


**Supplementary Figure 1.** The expression of five biomarkers in cluster A and B. (A) BIRC5, (B) KITLG, (C) PGF, (D) SHC1, (E) SPP1. *** represents P <0.001.
